# A novel nitro-dexamethasone inhibits agr system activity and improves therapeutic effects in MRSA sepsis models without antibiotics

**DOI:** 10.1038/srep20307

**Published:** 2016-02-03

**Authors:** Yun Yang, Haibo Li, Hongwu Sun, Li Gong, Ling Guo, Yun Shi, Changzhi Cai, Hao Gu, Zhen Song, Liuyang Yang, Yanan Tong, Chao Wei, Quanming Zou, Hao Zeng

**Affiliations:** 1 National Engineering Research Center of Immunological Products & Department of Microbiology and Biochemical Pharmacy, College of Pharmacy, Third Military Medical University, Chongqing 400038, PR China; 2 Department of Medicinal Chemistry, College of Pharmacy, Third Military Medical University, Chongqing 400038, PR China; 3 Institute of Immunology of PLA, Third Military Medical University, Chongqing 400038, PR China

## Abstract

Methicillin-resistant *Staphylococcus aureus* (MRSA) sepsis is a life-threatening medical condition that involves systemic inflammation throughout the body. Glucocorticoids are widely used in combination with antibiotics in the treatment of MRSA sepsis to fight the overwhelming inflammation. Here, we describe the improved anti-inflammatory properties of a nitric oxide (NO)-releasing derivative of dexamethasone, ND8008. ND8008 affected MRSA biofilm formation, caused biofilm cell death, and reduced the effects of virulence factors, such as α-toxin, by inhibiting the activity of the *Staphylococcus aureus* accessory gene regulator (agr) system. Dosing of mice with ND8008 (127.4 nmol/kg, i.p.) alone greatly reduced the inflammatory response caused by MRSA blood stream infection and considerably increased the survival rate of septic mice. These findings suggest that this novel NO-releasing derivative of dexamethasone ND8008 could be helpful in the treatment of MRSA sepsis.

Methicillin-resistant *staphylococcus aureus* (MRSA) sepsis is a blood infection with staphylococcus bacteria that are resistant to treatment with beta-lactam antibiotics. MRSA sepsis is a life-threatening medical condition that is caused by an overpowering immune response to MRSA infection and leads to systemic inflammation. Early diagnosis and rapid treatment increase the chances of patient survival, although the death rate from MRSA sepsis remains greater than 20% due to uncontrolled inflammation and drug resistance. These challenges necessitate investigations of new therapeutic approaches for MRSA sepsis.

As an anti-inflammatory medication, dexamethasone has shown a beneficial effect on the adjunct therapies of experimental staphylococcal endophthalmitis^1^,^2^, septic arthritis^3^, septic endocarditis^4^, and septic nephritis^5^. Recent studies have also indicated that the clinical use of corticosteroids in sepsis can restore cardiovascular homeostasis^6^, terminate systemic and tissue inflammation^7^, restore organ function, and prevent death^8^,^9^.

Recently, a series of investigations evaluated the therapeutic potential of such compounds in which a nitric oxide (NO)-releasing group was linked to well-established parent molecules^10^,^11^. NO-releasing glucocorticoid derivatives have shown an improved profile of pharmacological activity in terms of either enhanced anti-inflammatory efficacy or reduced side effects^12^,^13^. NO also represents an excellent antibacterial candidate because it is involved in the inhibition of bacterial respiration^14^ and DNA replication^15^,^16^. NO has been shown to be capable of inducing the dispersal of MRSA biofilm^17^,^18^,^19^, which is considered a major virulence factor due to the protective exopolysaccharide matrix that is resistant to penetration by antibiotics^20^. Moreover, NO plays a critical role in the host innate immune response to various bacterial infections^21^,^22^. We hypothesized that NO-releasing dexamethasone, compared with dexamethasone, would exert better effects on MRSA sepsis because it may possess improved anti-inflammatory and antibacterial activity.

A novel NO-releasing derivative of dexamethasone (ND8008) was synthesized in this study, and the protective effect of ND8008 in a model of MRSA sepsis was assessed without the use of antibiotics. In addition to evaluating anti-inflammatory and antibacterial activity, the underlying mechanisms of ND8008 on MRSA sepsis were investigated.

## Results

### Synthesis and characterization of ND8008

**Figure 1:**
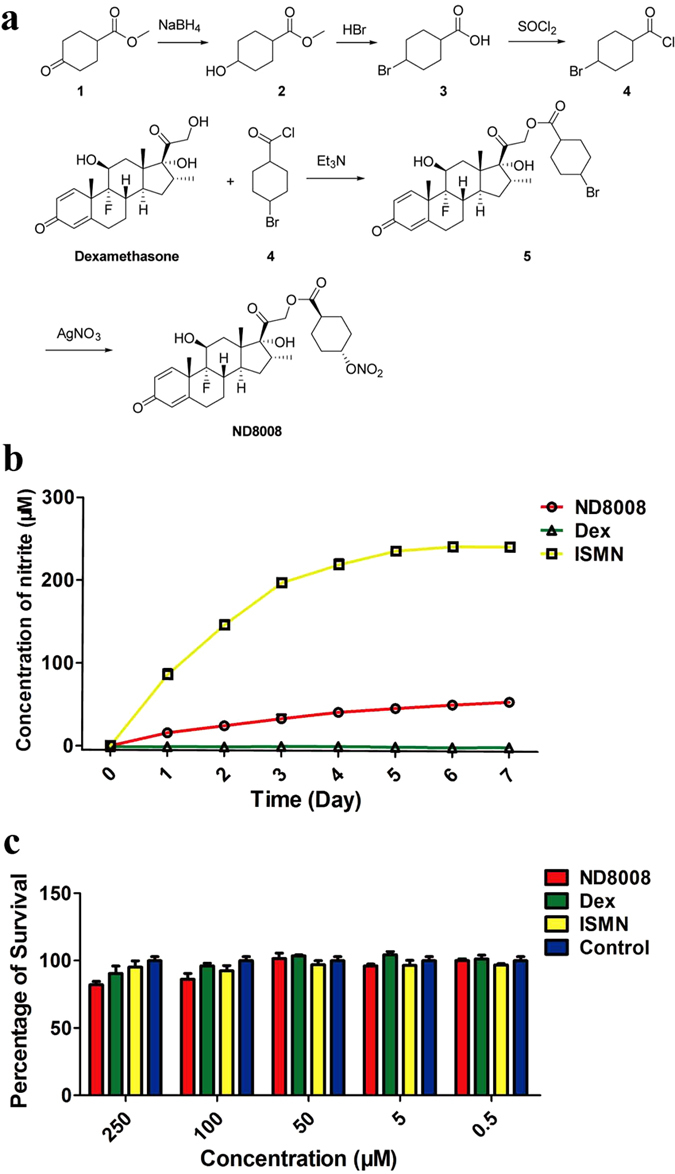
Synthesis and characterization of ND8008. (**a**) Chemical synthesis of ND8008. (**b**) The NO release rate of ND8008 *in vitro* was analysed by the Griess assay. 5-isosorbide mononitrate (ISMN) was used as a positive control, and dexamethasone (Dex) was used as a negative control. (**c**) Compound toxicity on murine peritoneal macrophages. For the Control, the same volume of ethanol was added to saline.

Previous studies have shown that dexamethasone 21-hydroxy ester-modification does not affect the glucocorticoid receptor in terms of ligand recognition^12^, nor influence the anti-inflammatory activity of the compound. ND8008 was prepared for the first time by incorporating an organic nitrate at the 21 position of dexamethasone through a *tans*-cyclohexanecarboxyate ester (Fig. 1a). Synthesis of ND8008 was initiated with the reduction of methyl 4-oxocyclohexanecarboxylate (**1**) followed by reaction with concentrated hydrobromic acid to give 4-bromo-cyclohexanecarboxylic acid (**3**), which was then converted to the corresponding acyl chloride (**4**) by treatment with refluxing thionyl chloride. After esterification of dexamethasone with **4**, ND8008 was eventually obtained by reaction with **5** and AgNO_3_. The steric configuration of the substituent cyclohexane in ND8008 was determined by comparing the melting point of ND8008-derived 4-hydroxycyclohexanecarboxylic acid, which was obtained by reduction removal of nitrate ester and LiOH hydrolysis, with that of literature data. The structure of ND8008 was confirmed by ^1^H NMR, ^13^C NMR, HMBC and NOESY spectra, which are provided in the Supplementary material. To simulate *in vivo* NO release from ND8008 and isosorbide mononitrate (ISMN), L-cysteine was used as the reducing agent, whose concentration approximately equal to that (10–20 mM) of the sulfhydryl groups in human blood, in the *in vitro* NO release assay. The results showed that the release rate of NO from ND8008 was much slower than that from ISMN (Fig. 1b). Furthermore, when ND8008 was tested for its effect on mice peritoneal macrophages using a Cell Counting Kit-8 (CCK-8) assay, no toxicity to cells was detected at concentrations from 500 nM to 250 μM (Fig. 1c). When the concentration reached 500 μM or above, cytotoxicity was observed in both dexamethasone and ND8008 (data not shown).

### ND8008 was more effective than dexamethasone at reducing the LPS-induced inflammatory response in macrophages

**Figure 2:**
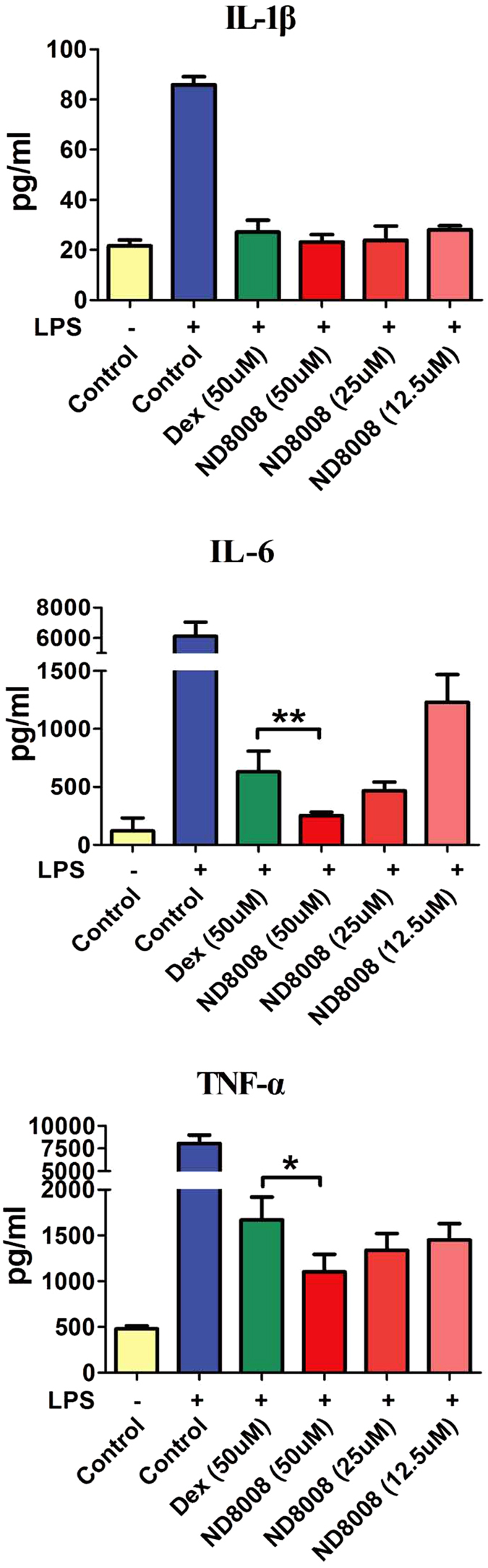
Effect of ND8008, Dex or ISMN treatment on cytokine levels of mouse peritoneal macrophages during lipopolysaccharide (LPS) exposure. Cells were pre-incubated with the compounds for 0.5 h and then stimulated with LPS (100 ng/mL) for 18 h. Significant differences between two groups were determined by Student’s t test. *p < 0.05 versus Dex (50 μM).

The *in vitro* anti-inflammatory effect of ND8008 was determined by a pro-inflammatory cytokine assay. Lipopolysaccharide (LPS) stimulation markedly increased the production of Tumor necrosis factor (TNF)-α, Interleukin (IL)-6 and IL-1β in the culture media supernatant of mice peritoneal macrophages (Fig. 2a–c). Although treatment with 50 μM dexamethasone significantly suppressed those increases, ND8008, at the same or lower concentration, suppressed the elevated levels of TNF-α and IL-6 to a greater extent than dexamethasone. IL-1β production was suppressed by ND8008 or dexamethasone to a level similar to that of the control group. Moreover, ND8008 suppressed acute pro-inflammatory cytokine production in a concentration-dependent manner.

### ND8008 had a slight inhibitory effect on MRSA growth

**Figure 3:**
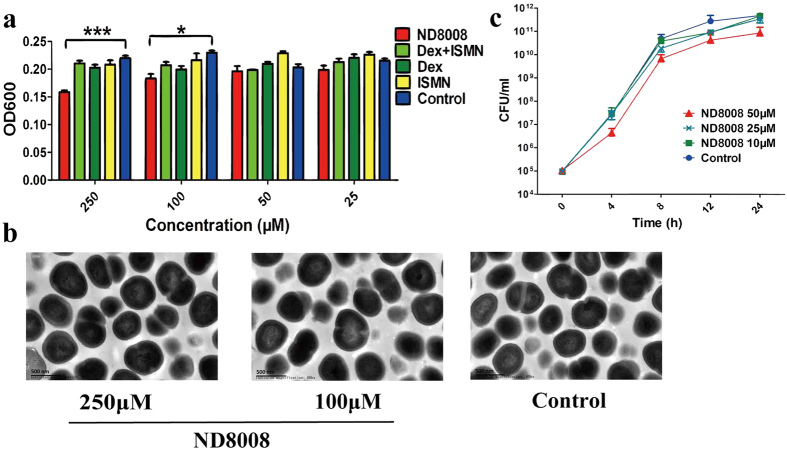
Effect of ND8008 on the growth and cell morphology of MRSA. (**a**) Growth at different concentrations of ND8008, Dex, ISMN or the mixture of dexamethasone and ISMN (Dex + ISMN) (10 to 250 μM) for 16 h in 96-well microtitre plates. Error bars indicate the standard deviation of 3 measurements. (**b**) TEM images of MRSA with 250/100 μM ND8008 treatment (at 16 h). (**c**) Growth at 10/25/50 μM ND8008 was monitored over time. ***P < 0.001 versus the control. *P < 0.05 versus the control. The same volume of ethanol was added to the control treatments in place of the drugs.

To evaluate the anti-MRSA effect of ND8008, growth of MRSA in the presence of varying concentrations of ND8008 was monitored by turbidity measurements after 16 h of incubation. As shown in Fig. 3a, ND8008 at high concentrations (≥100 μM) exhibited a slight inhibitory effect on MRSA growth. To determine the action mechanism of ND8008 on MRSA, morphological changes in the treated MRSA strains were investigated by transmission electron microscope (TEM) (Fig. 3b). No ultrastructural changes in the bacterial cell walls and membranes were found after MRSA exposure to ND8008 at inhibitory concentrations. Furthermore, ND8008 at concentrations of 50 μM, 25 μM and 10 μM did not affect the growth rate of MRSA (Fig. 3c). Therefore, these concentrations were adequate for performing the biofilm analysis.

### ND8008 inhibited MRSA biofilm formation and induced cell death within biofilms

**Figure 4:**
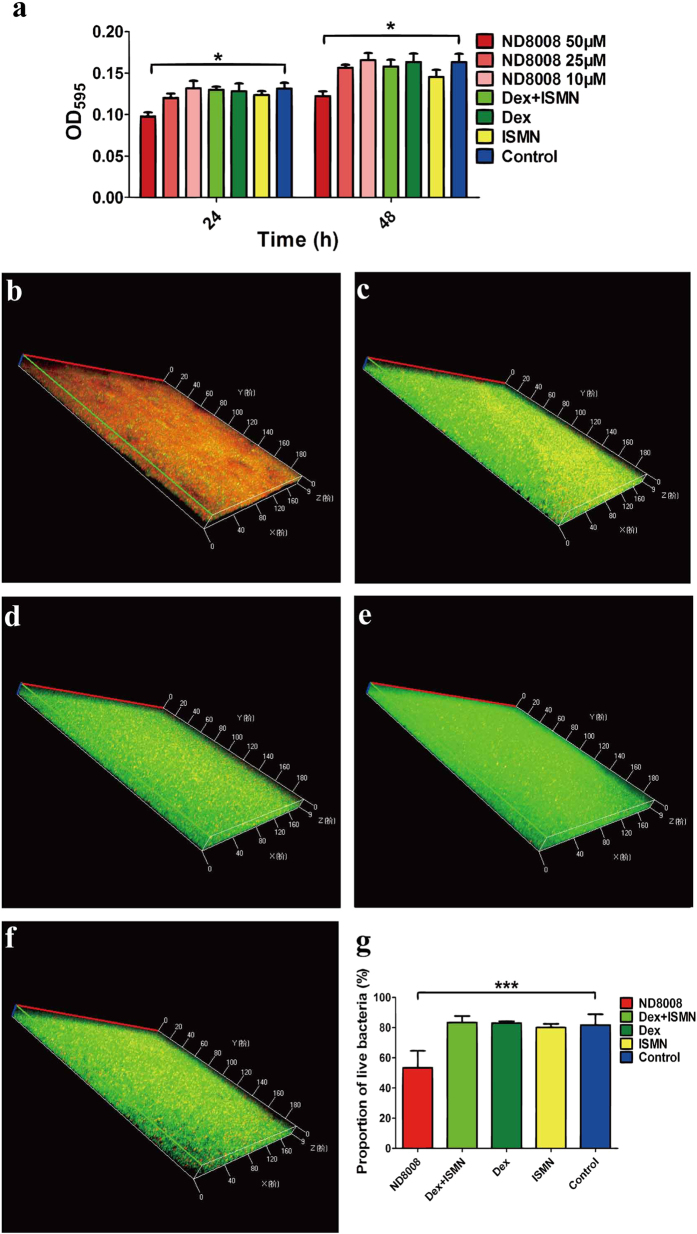
Effect of ND8008 on MRSA biofilm formation. (**a**) Crystal violet staining for biofilm quantity. MRSA biofilms were formed on 24-well microtitre plates and then washed and incubated for 24 h and 48 h with 10/25/50 μM ND8008, 50 μM Dex, 50 μM ISMN or 50 μM Dex + ISMN. CLSM images of MRSA with 50 μM ND8008, 50 μM Dex, 50 μM ISMN or 50 μM Dex + ISMN treatment. For confocal microscopy, MRSA biofilms were formed on plastic coverslips and then incubated for 24 h with 50 μM ND8008, Dex, ISMN or Dex + ISMN. The coverslips were then washed and analysed. The biofilms were stained with SYTO9 (green) for live cells and PI (red) for dead cells and extracellular DNA. (**b–f**) The three-dimensional structural images of biofilms treated with 50 μM ND8008 (**b**), 50 μM Dex (**c**), ISMN (**d**) or 50 μM Dex + ISMN (**e**). The same volume of ethanol was added to the control treatments in place of the drugs (**f**). (**g**) In the three-dimensional structural images of biofilms, the viable cells exhibited green fluorescence whereas, dead cells exhibited red fluorescence. The percentage of live bacteria relative to the total bacterial counts (shown in brackets) were calculated by using the ImageJ software. Error bars indicate the standard deviations of 4 measurements. ***P < 0.001 versus the control. *P < 0.05 versus the control.

**Figure 5:**
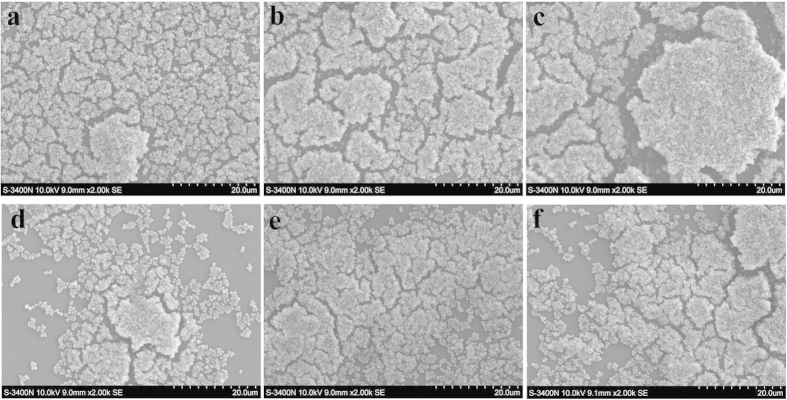
Scanning electron microscopy images showing the structure of methicillin-resistant *Staphylococcus aureus* under different conditions after 24 h. MRSA biofilms were formed on plastic coverslips and incubated for 24 h with different concentrations of ND8008, Dex or ISMN. The chips were then washed and analysed. (**a**) Control, (**b**) 50 μM dexamethasone, (**c**) 50 μM ISMN, (**d**) 50 μM ND8008, (**e**) 25 μM ND8008, (**f**) 10 μM ND8008. The same volume of ethanol was added to the controls in place of the drugs.

Recent evidence has shown that NO has effects on biofilm formation in a wide range of bacteria^23^, including *S. aureus*^24^. To establish the potential anti-biofilm activity of ND8008, crystal violet staining was used to semi-quantitatively analyse MRSA252 biofilm formation under different concentrations of ND8008 (10/25/50 μM). As shown in Fig. 4a, the concentration-dependent inhibition of biofilm formation was observed in wells treated with ND8008 after 24 h and 48 h of incubation. The anti-biofilm activity was further confirmed through confocal laser scanning microscopy (CLSM) and scanning electron microscopy (SEM) imaging. CLSM images demonstrated that treatment with 50 μM ND8008 resulted in more sparse distribution of biofilm than the controls. Moreover, ND8008 treatment significantly increased the proportion of dead cells within biofilms (Fig. 4b–g). At the same concentration, ISMN, dexamethasone or the mixture of them had no such effect. The SEM images also demonstrated that the biofilm structure after exposure to ND8008 (10/25/50 μM) was significantly disrupted and showed a much lower density of bacterial cell clusters than the controls (Fig. 5a–f).

### ND8008 treatment inhibits the activation of the MRSA agr system

**Figure 6:**
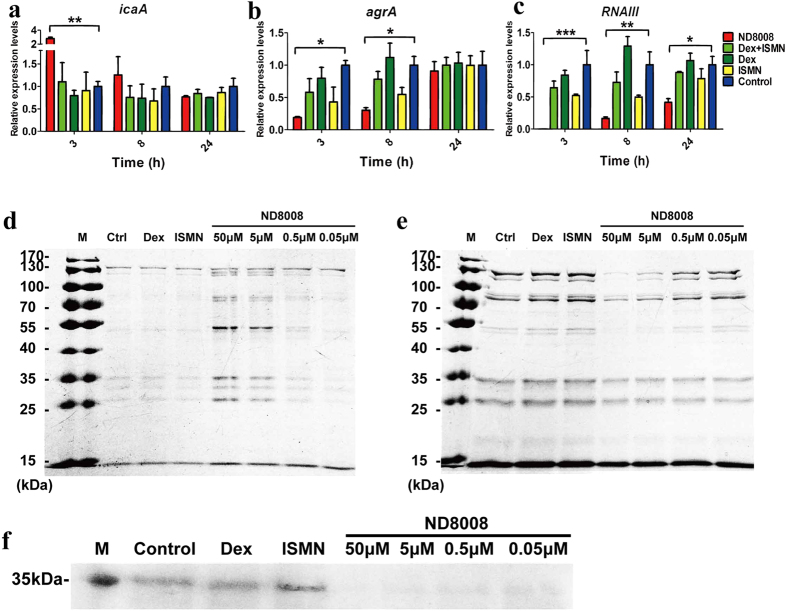
Treatment of MRSA with ND8008 inhibits the activation of agr and RNAIII. *In vitro* expression of *icaA* (**a**), *agrA* (**b**) and *RNAIII* (**c**) in MRSA studied at 3, 8 and 24 h of growth with 50 μM ND8008, Dex, ISMN or Dex + ISMN treatment. Data were obtained by RT-PCR, and the relative transcript levels of *agrA, icaA* and *RNAIII* represent the mean (±standard deviations) of at least two biological replicates. The relative magnitude of the gene expression level was defined as the copy number of cDNA of each gene in the MRSA cells normalized by the copy number of cDNA of the corresponding gene in cells of the control group. SDS-PAGE analysis of MRSA surface proteins (**c**) and secretory proteins (**d**) after treatment with 50 μM ND8008, Dex or ISMN. (**e**) Immunoblot for alpha hemolysin in the supernatants from the MRSA cells with 50 μM ISMN, Dex or 50 μM, 5 μM, 0.5 μM or 0.05 μM ND8008. **P < 0.01 versus the control (Ctrl). *P < 0.05 versus the control. The same volume of ethanol was added to the controls in place of the drugs.

Polysaccharide intercellular adhesion (PIA), encoded by the *icaADBC* locus, is a major matrix component of *S. aureus* biofilms^25^. We subsequently analysed the expression level of *icaA*. Only ND8008 treatment up-regulated the expression at 3 h of growth; no difference was observed in any group during any other period of growth (Fig. 6a).

The agr quorum-sensing system has been shown to regulate *S. aureus* biofilm formation^26^. We analysed the transcriptional levels of *agrA* and *RNAIII* to determine whether the transcriptional levels of these genes were associated with the reduction of biofilm formation when ND8008 was added. The *agrA* gene was significantly down-regulated in MRSA252 cells treated with 50 μM ND8008 at 3 h and 8 h of growth, whereas ISMN and Dex + ISMN treatment appeared to down-regulate the *agrA* gene in the same period, although to a lesser extent than ND8008 treatment (Fig. 6b). Surprisingly, there was a 316-fold reduction in *RNAIII* expression after 3 h of growth in the ND8008 treatment, and this significant effect continued at 24 h of growth. Although ISMN and Dex + ISMN treatment could down-regulate the expression of *RNAIII*, this effect was much weaker than ND8008 and was observed only after 3 h and 8 h of growth (Fig. 6c).

*RNAIII* is known to control the expression of a large number of virulence genes such as those for α-toxin^27^ and δ-toxin^28^, and down-regulation of the expression of *RNAIII* may inhibit the production of these exotoxins. SDS-PAGE analysis showed that ND8008 treatment increased the expression of MRSA surface proteins (Fig. 6d) and reduced the expression of excreted proteins (Fig. 6e). Immunoblot assays showed the presence of α-toxin in the supernatants of MRSA grown in the absence of ND8008, although the level was substantially diminished in the presence ND8008 in a concentration-dependent manner (Fig. 6f).

### ND8008 reduced the systemic inflammatory response in a sub-lethal MRSA sepsis model but did not affect the bacterial load

**Figure 7:**
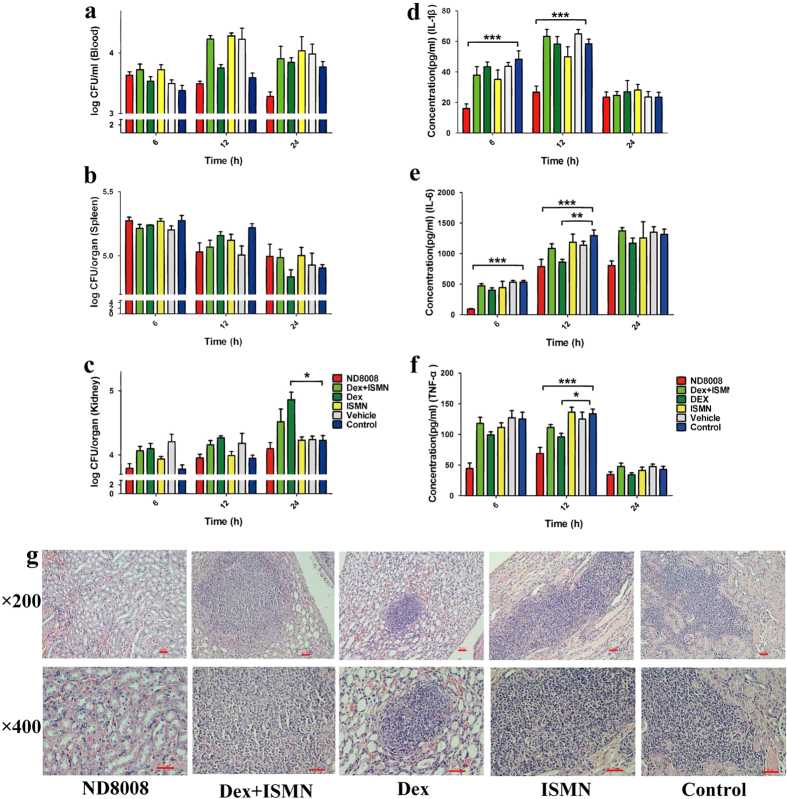
**ND8008 had a potent anti-inflammatory activity** ***in vivo*** **but did not affect the bacterial load in organs.** (**a–c**) Effects of ND8008, Dex, ISMN or Dex + ISMN treatment on MRSA bacterial clearance in the plasma (**a**), spleen (**b**) and kidney homogenates (**c**) at 3, 6 and 12 h following inoculation. (**d–f**) Effect of ND8008, Dex, ISMN or Dex + ISMN treatment on cytokine levels during MRSA sepsis, respectively, for IL-1β (**d**), IL-6 (**e**) and TNF-α (**f**) levels in plasma obtained at 3, 6 and 12 h after bacterial inoculation. Data are shown as the mean ± SEM of 6 animals per group. *P < 0.05 versus the control. ***P < 0.001 versus the control. (**g**) Hematoxylin & eosin staining of kidneys from mice with ND8008, Dex, ISMN or Dex + ISMN treatment. Three days after inoculation, the kidneys were harvested and stained. Representative histopathological sections from 5 mice per group are shown (magnification = 200× (upper panels) and 400× (lower panels).

We used a sub-lethal mouse sepsis model to investigate the therapeutic effect of ND8008. Mice were inoculated intravenously with 3 × 10^8 ^CFU/mouse of MRSA252, and they then received ND8008 (127.4 nmol/kg) treatment without antibiotics. Bacterial colonization in the host tissue, including the blood, kidneys and spleen, was not affected by ND8008 treatment. In contrast, the bacterial load in the host kidneys was significantly increased by dexamethasone treatment 24 h after inoculation (Fig. 7a–c).

It is believed that the levels of pro-inflammatory cytokines including IL-1β, TNF-α and IL-6 strongly correlate with the extent and severity of MRSA sepsis. The pro-inflammatory marker IL-1β was significantly decreased in the ND8008 treatment group compared to the control group, and the decrease was more pronounced than that in the dexamethasone group (Fig. 7d). For IL-6 and TNF-α, we got similar results (Fig. 7e–f).

Kidney abscesses are a hallmark of a MRSA bloodstream infection. Hematoxylin-Eosin (HE) stained sections were used to investigate the histopathological alterations by light microscopy. The histological analysis showed that the kidneys of mice treated with ND8008 had no abscesses 3 days after inoculation (Fig. 7g).

### ND8008 treatment improved survival in a lethal MRSA sepsis model without antibiotics

**Figure 8:**
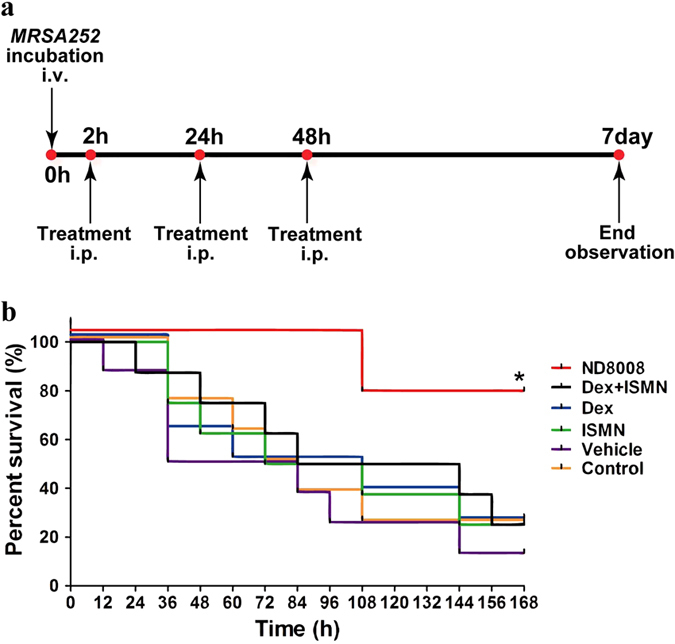
ND8008 treatment improved survival in a lethal MRSA sepsis model. (**a**) Schedule for drug administration and measurement of the survival rates up to 7 days after inoculation. (**b**) Effect of 127.4 nmol/kg ND8008, Dex, ISMN or Dex + ISMN treatment on septic mouse survival. ND8008 treatment was associated with improved survival compared with saline treatment (Control, P = 0.0180), vehicle (the same volume of ethanol added to saline as vehicle, P = 0.0040), Dex (P = 0.0305), ISMN (P = 0.0294) or Dex + ISMN (P = 0.0380). The significance was determined using the log rank test. *P < 0.05 versus the control.

Next, we investigated whether ND8008 treatment was beneficial for a mouse lethal MRSA sepsis model without using any antibiotics. Mice were inoculated intravenously with 1 × 10^9 ^CFU/mouse of MRSA252 and received 127.4 nmol/kg of ND8008, dexamethasone, ISMN or Dex + ISMN 2 hours after inoculation for three consecutive days, which was administered by intraperitoneal injection (Fig. 8a). Vehicle group mice received saline containing 1% alcohol, and the control group received no special treatment. In the control group, MRSA252 resulted in an overall survival of 25% at the 7th day following infection. Dexamethasone, ISMN and Dex + ISMN were unable to prevent lethal infection by MRSA. Remarkably, ND8008 treatment significantly improved survival, which was 75% on day 7 post inoculation (Fig. 8b).

## Discussion

NO is a messenger or an effector molecule in the cardiovascular, nervous and immune systems and has many other influential functions^29^,^30^,^31^. The design and study of NO-donating drugs has become an important strategy in innovative drug research. Here, we describe the therapeutic effects of a nitro-dexamethasone, ND8008, in an experimental model of MRSA sepsis.

NO has been found to exhibit antimicrobial activity against MRSA in many studies. As a NO donor, ND8008 had a slight inhibitory effect on the growth of MRSA at high concentrations (≥100 μM). Ultrastructural studies revealed intact cells after exposure to ND8008 at inhibitory concentrations, indicating that the anti-MRSA mechanism of ND8008 is independent of cell lysis. Given that ND8008 exhibits weak anti-MRSA activity in concentrations much higher than those we used in treatment of animals, we deduced that the inhibitory effect of ND8008 on MRSA was not the essential protective factor for MRSA infection in this study.

NO can inhibit microbial biofilm formation, and thus, the inhibitory effect of ND8008 on MRSA biofilm formation was studied. We found that at 50 μM, neither ISMN nor dexamethasone had an inhibitory effect on MRSA biofilm formation, even the mixture of them; however, ND8008 at 50 μM had significant inhibitory effect. Using SEM, we found that the structure of ND8008-treated MRSA biofilm was disrupted and bacterial cell clusters were less dense than the controls. Subsequently, we used LCMS to observe biofilm cells with LIVE/DEAD labelling. The proportion of dead cells within ND8008-treated MRSA biofilm was much higher than that in the dexamethasone, ISMN and control groups, indicating that ND8008 may effectively lead to MRSA cell death within biofilms by exerting an inhibitory effect. The NO donor ISMN was reported to have an inhibitory effect on the growth of MRSA^24^ but requires a much higher concentration than that used in this experiment. NO^32^ and corticosteroids^33^ can reduce MRSA biofilm formation at high concentrations, although these mechanisms remain unclear. Given that the ND8008 had anti-biofilm activity at the lower concentration, we hypothesized that this effect was derived from chemical coupling of dexamethasone and the nitrate.

The mechanisms involved in MRSA biofilm formation are multifaceted and complicated*. IcaA* is involved in the synthesis of PIA, and the latter forms a complex extracellular matrix and mediates MRSA biofilm accumulation^34^. ND8008 treatment did not affect the expression level of *IcaA* in the mid- or late-exponential growth phase but increased the expression of *icaA* in the early-exponential growth phase. The *icaA* level did not seem to be associated with the inhibitory effect of ND8008 on MRSA biofilm formation. The influence of the agr system on biofilm formation remains controversial. Although evidence suggests that the inhibition of the agr system is important for biofilm formation^26^, other studies suggests that the agr system divergently regulates biofilm formation^35^. In the current study, ND8008 treatment significantly inhibited the agr system, which was characterized by significantly decreased expression of *agrA* with dispersal of biofilm; thus, it was unclear whether the down-regulation of the agr system was involved in the anti-biofilm effect of ND8008.

RNAIII is the intracellular effector of the agr quorum sensing system in *S. aureus*. It is one of the largest regulatory RNAs (514 nucleotides long) known to control the expression of a large number of virulence genes such as α-toxin^27^ and δ-toxin^28^. It also influences the expression of a number of important virulence factors such as toxic shock syndrome toxin-1 (TSST-1)^36^ and multiple enterotoxins^37^. α-toxin simultaneously alters platelet activation and promotes neutrophil inflammatory signalling; platelet and immune cell activation contribute to organ injury during sepsis and increase mortality^38^. ND8008 treatment markedly decreased *RNAIII* transcription. As a result, α-toxin production was significantly diminished by ND8008 even at 0.5 μM, which may greatly contribute to mouse survival in lethal sepsis models. It was also reported that inhibition of RNAIII by a heptapeptide can significantly reduce *S. aureus* biofilm formation *in vitro* and *in vivo*^39^,^40^. α-toxin is required for biofilm formation^41^; thus, we speculated that the inhibitory effect of ND8008 on MRSA biofilm formation may be associated with the inhibition of *RNAIII* expression and the diminishment of α-toxin production.

Previous studies revealed that a low dose of dexamethasone (130 nmol/kg) administered early (2 hours after infection) during the treatment of sepsis induced by *Staphylococcus aureus* has a good protective effect in combination with antibiotics^42^,^43^. With regards to the inhibition of MRSA biofilm formation and interference with the agr system by ND8008, the therapeutic effect of ND8008 was investigated in a sub-lethal MRSA sepsis model without using antibiotics. Our data showed that NO can synergize with the dexamethasone moiety to produce a more potent anti-inflammatory effect. We found that low-dose ND8008 therapy could significantly reduce the initial infection, especially 6–12 hours later in the host serum pro-inflammatory cytokine (IL-1, IL-6 and TNF-α) content. ND8008 also exhibited a better anti-inflammatory effect than dexamethasone and did not cause an increase in the bacterial load in organs, which indicated that this dose of ND8008 could effectively alleviate inflammation without causing excessive immunosuppression. Both NO^44^ and dexamethasone^45^ inhibited IL-1 converting enzyme expression; the potent inhibitory effect of ND8008 on IL-1β may benefit from synergy between NO and dexamethasone. In contrast, the same dose of dexamethasone had a mild anti-inflammatory effect, and 24 hours after infection challenge in mice there was a greater amount of bacterial colonization in the kidneys than in other groups, which suggests relatively significant immunosuppressive effects. ND8008-treated mice had no abscess and inflammatory cell infiltrates in the kidneys, further implicating its excellent anti-inflammatory effects. The collective evidence indicates that ND8008 exerts beneficial effect on MRSA infection.

We then evaluated the protective effect of ND8008 in a lethal MRSA sepsis model without antibiotics, and found that ND8008 had an excellent protective effect, and could reduce mortality. Mice receiving the higher dose of ND8008 (2.55 μmol/kg) were not well protected due to the immunosuppressive effects of dose escalation without the use of antibiotics for elimination of pathogenic microorganisms in the host (data not shown). However, the mixture of the same dose of dexamethasone and ISMN did not provide protective effect, indicating that the protection occurs via a chemical coupling t of dexamethasone in combination with the nitrate.

ND8008 treatment remarkably reduced the secretion of exotoxins such as α-toxin, which in a sense also contributed to the synergistic anti-inflammatory effect. This may also explain why the difference in anti-inflammatory activity between ND8008 and dexamethasone *in vivo* was greater than *in vitro*; the inflammatory response induced by LPS instead of live bacteria was used in the *in vitro* activity detection. Furthermore, Dex + ISMN could not mimic the effect of ND8008 in most cases, and the possible reason was that NO release from ND8008 was much slower than that from ISMN. A previous study also reported that prednisolone synergism with a slower-releasing NO donor NOC-18 (DETA NONOate) exhibited a stronger anti-inflammation effect than synergism with sodium nitroprusside^46^. The relationship between the NO-release kinetic and the pharmacological activity will be further studied in the future.

In conclusion, the glucocorticoid derivative ND8008 has a more potent effect than dexamethasone on some of the measured inflammatory parameters. In addition, based on its strong adverse effects on MRSA biofilms and virulence, ND8008 may be an option for treating MRSA sepsis. More studies are urgently needed to confirm these findings detailed herein on the glucocorticoid derivative ND8008.

## Materials and Methods

### Chemical synthesis

The NO-releasing derivative of dexamethasone, ND8008, was prepared according to Fig. 1a, using protocols described in the Supplementary material. ^1^H NMR, ^13^C NMR, HMBC and NOESY spectra for ND8008 are provided (Supplementary Fig. S1–4).

### Bacterial strain and culture media

Methicillin-resistant *S. aureus* MRSA252 was obtained from the American Type Culture Collection (Manassas, USA). Tryptic soy broth (TSB) (OXOID, UK) supplemented with 0.5% glucose and 2.0% NaCl was used for biofilm production^47^. Time-kill tests and CFU counting were performed in Mueller-Hinton broth (AOBOX Biotechnology, China). Stocks were kept at −80 °C in Mueller-Hinton broth supplemented with 15% glycerol.

### Animals

Six- to eight-week-old SPF Male C57BL/6J mice were purchased from the Experimental Animal Center of Third Military Medical University. Animal maintenance and experimental procedures were performed in accordance with the National Institutes of Health Guidelines for the Use of Experimental Animals and were approved by the Medicine Animal Care Committee of the Third Military Medical University.

### NO release assay

The synthesized compound ND8008 and dexamethasone were dissolved in ethanol to a concentration of 5 mM. L-cysteine was then added to a final concentration of 50 mM in 5 mL of a 1:1 (v/v) methanol:PBS (pH 7.4) mixed solution. The drugs were added to the above methanol-PBS solution containing L-cysteine to a final concentration of 400 μM and incubated at 37 °C. At each time point, 50 μL was removed for the NO assay. ISMN was used as a positive control, and each assay was repeated three times. Nitrite was measured by mixing 50 μL of culture supernatants with the same volume of Griess reagent (Sigma, USA) and reading the optical density at 550 nm after 15 min. A standard curve was prepared using 0 to 80 μM NaNO_2_ standards in double-distilled water (ddH2O).

### Cell viability

Peritoneal macrophages were isolated and purified from mice as previously described^48^. Viability assays were performed using a Cell Counting Kit-8 (Dojindo, Japan) in 96-well plates (Corning, USA). Each well was seeded with 1000 mice peritoneal macrophage cells in DMEM media and incubated at 37 °C inside a CO_2_ incubator for 24 h. The medium was replaced with 100 μL of fresh culture medium with dexamethasone, ethanol, ISMN, Dex + ISMN or ND8008, and the plates were then incubated for an additional 24 h at 37 °C. The medium was then removed, and 100 μL of DMEM media supplemented with 10 μL of CCK-8 was added to each well. After 4 h of incubation at 37 °C, the optical density of each well was measured at 450 nm.

### Lipopolysaccharide (LPS)-stimulated culture

Peritoneal macrophages were isolated and purified from mice as previously described^49^. Cells were grown and cultured in RPMI 1640 supplemented with 10% foetal bovine serum. A combination of 100 U/mL penicillin and 100 μg/mL streptomycin was added under 5% CO_2_ at 37 °C in humidified air. Cells were plated at 1.0 × 10^6 ^cells/mL in a plate with 96 wells (200 μL/well). The tested compounds were dissolved in DMSO and diluted with supplemented DMEM as needed. Cells were pre-incubated with the compounds for 0.5 h and then stimulated with LPS (100 ng/mL) for 18 h. Dexamethasone was used as positive control. For a negative control, no LPS was added. The levels of TNF-α, IL-6 and IL-1β in the supernatants were analysed using the appropriate ELISA kits (Bioscience, Germany).

### MRSA sepsis model and treatments

Mice were infected with 9 × 10^8 ^CFU/mouse of MRSA252 in 200 μL of PBS (LD90 suspension) via the tail vein for the lethal MRSA sepsis model^42^. The survival rates were monitored for 7 days after infection. On the eighth day post infection, mice were killed by CO_2_ asphyxiation. The non-lethal sepsis model was used to evaluate the anti-inflammatory and antibacterial effects of ND8008. Briefly, mice were infected with 1 × 10^8 ^CFU/mouse of MRSA252 in 200 μL of PBS via the tail vein, and the spleens and kidneys were harvested at 6 h, 12 h and 24 h for bacterial burden and blood cytokine assays. For both the lethal and non-lethal MRSA sepsis models, mice were administered intraperitoneally with ND8008 at 127.4 nmol/kg once daily. For comparison, infected mice were also treated with vehicle (the same volume of ethanol added to saline), dexamethasone, ISMN or Dex + ISMN. All of these reagents were administered for 3 consecutive days starting 2 h after MRSA inoculation.

### Bacterial burden, serum cytokines and histopathology

The blood, spleens, and kidneys were harvested at 6 h, 12 h and 24 h post infection for the determination of the bacterial burden in the non-lethal sepsis model. The bacterial load in the organs was evaluated by preparing organ homogenates in PBS and plating 10-fold serial dilutions on Muler-Hinton agar (AOBOX Biotechnology, China). The colonies were counted after 24 h of incubation at 37 °C. Blood cytokines were analysed by ELISA (Bioscience, Germany) according to the manufacturer’s instructions. For histopathology, the kidneys were harvested on day 3 post infection and fixed with 4% paraformaldehyde and embedded in paraffin. Four-micrometre-thick sections were prepared and stained with hematoxylin and eosin for microscopic examination.

### Growth curves

Growth curves for the drugs were obtained with a range of different concentrations containing a final inoculum of 10^5 ^CFU/mL of MRSA252. After 0, 2, 4, 8 and 24 hours of incubation, viability expressed as CFU/mL was determined by plating 100 μL of serial dilutions onto Mueller –Hinton agar plates. The CFUs were counted after incubation at 37 °C overnight.

### Crystal violet staining

An overnight culture of MRSA252 was diluted in TSB (0.5% glucose and 2% NaCl) to a final concentration of 1 × 10^7 ^CFU/mL. Biofilms were grown in clear flat-bottomed, tissue culture-treated 24-well microtitre plates at 37 °C. Supernatant was gently removed, and the wells were washed twice with 1 mL of PBS. The remaining biofilm was then stained with 100 μL of 0.5% CV dye, rinsed twice with 2 mL of ddH2O and thoroughly dried. For quantification, 1 mL of 30% acetic acid was added to each well. Plates were incubated for one hour at room temperature with shaking. The CV solution was diluted, and the OD at 570 nm was measured with a microplate reader.

### Confocal laser scanning microscopy (CLSM)

An overnight suspension of MRSA252 was diluted to a final concentration of 1 × 10^7 ^CFU/mL, and 2 mL of the diluted medium containing test compounds or the control was transferred to 24-well plates with each well containing a plastic coverslip (Thermo Scientific, USA). Biofilms were allowed to form for 24 h at 37 °C. After treatment removal, biofilms on the coverslips were stained with a Live/Dead BacLight^®^ bacterial viability kit (Molecular Probes, USA) according to the manufacturer’s specifications. Briefly, suspensions on the coverslips were gently aspirated, washed once with saline and treated with Live/Dead BacLight^®^ SYTO 9 and propidium iodide stains for 15 min at room temperature. After staining, the coverslips were washed an additional two times with 1 mL of ddH_2_O. Coverslips were mounted on a microscope slide and immediately observed using a LSM 780 confocal laser scanning microscope (CLSM, Zeiss, Germany).

### Scanning electron microscopy (SEM)

A 2 mL suspension of MRSA252 (1 × 10^7 ^CFU/mL) containing 50 μM ND8008, 50 μM dexamethasone, 50 μM ISMN, the mixture of 50 μM dexamethasone and 50 μM ISMN or the control was transferred to 24-well plates with each well containing a plastic coverslip (Thermo Scientific, MA, USA). Biofilms were allowed to form for 24 h at 37 °C. After treatment removal, biofilms on plastic coverslips were processed for SEM by dehydration in a graded concentration series of ethanol. Samples were sputter-coated with 200 Å of gold-palladium and viewed in a S-3400N scanning electron microscope (Hitachi, Japan) with an accelerating voltage of 10 kV.

### Transmission electron microscopy (TEM)

Exponential phase cultures of MRSA252 (1 × 10^5 ^CFU/mL) were amended with 50 μM ND8008, dexamethasone, ISMN or the same volume of ethanol as a control. Samples were then incubated for 24 h with shaking in MHB prior to centrifugation at 3000 rpm for 10 min to pellet the cells. The supernatant was discarded, and the cells were washed three times with PBS prior to treatment for observation using an FEI TECNAI10 transmission electron microscope (Philips Electron Optics, Holland).

### Quantitative RT-PCR

The expression levels of *agrA, RNA III* and *icaA* were analysed by quantitative RT-PCR^50^. Briefly, overnight cultures of MRSA252 were diluted 100-fold in fresh TSB medium and grown at 37 °C in a shaking incubator. Cells were harvested after 3, 8 and 24 h of growth, representing the mid-exponential phase, the late exponential phase, and the stationary phase, respectively. Quantitative real-time PCR was performed using an ABI Prism 7000 instrument (Applied Biosystems, USA) and the SYBR green PCR master mix (TOYOBO, Japan). Reaction mixtures were prepared using the primers listed in Supplementary Table S1 at 100 nM. A well-characterized gene, *gyrB*, was used as an internal control. Relative target gene expression was calculated as the differences in cycle thresholds (DCT) (*gyrB* CT–target gene CT) for all samples. qRT-PCR experiments were performed using at least two biological replicates, with each tested in triplicate.

### MRSA surface and secreted protein extraction and processing

MRSA252 was grown overnight in TSB broth. Cultures were then diluted 1:100 with TSB broth containing 50 μM ND8008, dexamethasone or ISMN or ethanol (1%) as a control. Cultures were then incubated for 15 h at 37 °C with 160 rpm shaking. Subsequently, cultures were diluted with TSB to the same OD600, centrifuged, and washed 3 times with PBS. The surface proteins were isolated and run on an SDS-PAGE gel according to a previously published protocol^51^. Preparation of MRSA252 culture supernatant was performed as previously reported^49^. Briefly, supernatants were filtered through a 0.22 mm filter and concentrated with Amicon Ultra 10 kDa nominal molecular weight limit centrifugal filter units (Millipore, USA). Secreted proteins in culture supernatants were resolved by SDS-PAGE after the addition of a quarter volume of 5 × SDS buffer and heating to 95 °C for 10 minutes. The resolved proteins were then transferred to a nitrocellulose membrane and subjected to immunoblotting using a rabbit polyclonal anti-Hla antibody (Abcam, USA). An HRP-labelled secondary antibody was used for visualization.

### Statistical analysis

Statistical analysis was performed using the Graphpad Prism program (version 5.01). Statistical significance was designated on the graphs using asterisks (*, **, or***) for P values less than 0.05, 0.01, and 0.001, respectively. Student’s t test was used for comparison of two data sets. For more than two sets of data, the ANOVA test was performed, followed by Tukey’s test.

## Additional Information

**How to cite this article**: Yang, Y. *et al*. A novel nitro-dexamethasone inhibits agr system activity and improves therapeutic effects in MRSA sepsis models without antibiotics. *Sci. Rep*. **6**, 20307; doi: 10.1038/srep20307 (2016).

## Supplementary Material

Supplementary Information
