# Supplementary material for: A novel nitro-dexamethasone inhibits agr system activity and improves therapeutic effects in MRSA sepsis models without antibiotics
Source: Sci Rep. 2016 Feb 3;6:20307. doi: 10.1038/srep20307 (PMC4738243; doi:10.1038/srep20307)
Supplement: Supplementary Information [file srep20307-s1.doc]

**Supplementary material**

**A novel nitro-dexamethasone inhibits agr system activity and improves therapeutic effects in MRSA sepsis models without antibiotics**

Yun Yang^1*^, Haibo Li^2*^, Hongwu Sun^1^, Li Gong^1^, Ling Guo^3^, Yun Shi^1^, Changzhi Cai^1^, Hao Gu^1^, Zhen Song^1^, Liuyang Yang^1^, Yanan Tong^1^, Chao Wei^1^, Quanming Zou^1^ & Hao Zeng^1^.

^1^National Engineering Research Center of Immunological Products & Department of Microbiology and Biochemical Pharmacy, College of Pharmacy, Third Military Medical University, Chongqing 400038, PR China

^2^Department of Medicinal Chemistry, College of Pharmacy, Third Military Medical University, Chongqing 400038, PR China

^3^Institute of Immunology of PLA, Third Military Medical University, Chongqing 400038, PR China.

*These authors contributed equally to this work.

**Correspondence：**

Prof. Hao Zeng and Prof. Quanming Zou, National Engineering Research Center of Immunological Products, Department of Microbiology and Biochemical Pharmacy, College of Pharmacy, Third Military Medical University, Chongqing 400038, PR China.

**Phone & Fax:** 86-023-68752376 (Prof. Hao Zeng); 86-023-68752375 (Prof. Quanming Zou)

**Email:** Zeng1109@163.com (Prof. Hao Zeng); qmzou2007@163.com (Prof. Quanming Zou)

SUPPLEMENTARY METHODS.....................................................................................................3

SUPPLEMENTARY FIGURES........................................................................................................6

SUPPLEMENTARY TABLES .........................................................................................................9

**1 Supplementary Methods**

**Chemical Syntheses**

**General.** Unless otherwise noted, materials were purchased from commercial suppliers and were used as received. Solvents used for chromatography were distilled prior to use. All anhydrous reactions were carried out under a nitrogen atmosphere using oven-dried glassware. ^1^H and ^13^C nuclear magnetic resonance (NMR) spectra were recorded on an Agilent 600 MHz NMR spectrometer. ^1^H NMR, ^13^C NMR, HMBC and NOESY spectra are reported in parts per million (ppm) downfield from an internal standard, tetramethylsilane (0 ppm) and CHCl_3_ (77.0 ppm), respectively.

**Methyl 4-hydroxyl-cyclohexanecarboxylate (2):** NaBH_4_ (19.0 g, 500 mmol) was added in small portions to a solution of methyl 4-oxocyclohexanecarboxylate (**2**) (31.2 g, 200 mmol) in MeOH (400 mL) at 0°C, and the mixture was then stirred at room temperature for 3 h. The reaction was quenched by the addition of H_2_O (500 mL) and extracted with DCM (300 mL × 3). The combined organic layer was washed with H_2_O (500 mL), dried over Na_2_SO_4_, and concentrated *in vacuo*. The residue was purified by vacuum distillation to give **2** (28.8 g, 91.1%) as a clear oil.

**4-bromo-cyclohexanecarboxylic acid (3):** **2** (28.8 g, 18.2 mmol) was mixed with 250 mL of concentrated HBr and stirred at 50°C for 24 h. The reaction was cooled and then extracted with DCM (100 mL × 4). The combined organic layer was washed with H_2_O, saturated NaCl, dried over Na_2_SO_4_, and concentrated under reduced pressure. To the solution of the residue in ethyl acetate (30 mL) at 0°C, 250 mL of petroleum ether was added dropwise and stirred for another 5 h. Product **3** (19.2 g, 50.9%) was filtered off as a white solid.

**(11β,16α)-1,4-diene-9-fluoro-11,17-dihydroxy-16-methyl-21-[[4-bomo-cyclohexanecarboxyl]oxy] pregna 3,20-dione (5):** **3** (1.92 g, 9.275 mmol) was placed in a dried round-bottomed flask, and SOCl_2_ (10 mL) was added dropwise with stirring. The mixture was heated under reflux for 5 h, and the excess SOCl_2_ was then removed. Product **4** (1.54 g, 73.65%) was separated by fractional vacuum distillation. Dexamethasone (11.172 g, 28.5 mmol) was dissolved in 150 mL of absolute tetrahydrofuran (THF) and cooled to 0°C; **4** (42.75 mmol) and triethylamine (85.5 mmol) were added by syringe under a protective atmosphere of argon. The reaction mixture was allowed to warm to room temperature and stirred continuously overnight. The solvent was evaporated under reduced pressure, and the residue was washed three times with petroleum ether/ethyl acetate (300 mL, 1/10, v/v). After filtration and evaporation of the solvent, the residue was separated by chromatography on silica gel to give **5** (13.6 g, 73.65%).

**(11β,16α)-1,4-diene-9-fluoro-11,17-dihydroxy-16-methyl-21-[[*trans*-4-(nitrooxy)-cyclohexanecarboxyl]oxy] pregna 3,20-dione (ND8008):** **5** (13.6 g, 23.388 mmol) was dissolved in 150 mL of dry acetonitrile, and silver nitrate (7.95 g, 46.776 mmol) was added. The mixture was heated at 45°C for 8 h under a protective atmosphere of argon. After the solvent was removed under vacuum, the residue was purified by silica gel column chromatography to give 10.6 g ND8008 (76.3% yield). ^1^H NMR (600 MHz, CDCl_3_, Supplementary Fig. 1) δ 7.20 (d, *J* = 10.1 Hz, 1H), 6.32 (dd, *J* = 10.1, 1.5 Hz, 1H), 6.09 (s, 1H), 4.97 – 4.85 (m, 3H), 4.34 (d, *J* = 8.6 Hz, 1H), 3.07 (ddd, *J* = 11.1, 7.3, 3.9 Hz, 1H), 2.64 – 2.55 (m, 2H), 2.48 (tt, *J* = 10.6, 3.3 Hz, 1H), 2.44 – 2.30 (m, 3H), 2.22 – 2.06 (m, 6H), 1.92 (s, 2H), 1.86 – 1.61 (m, 6H), 1.58 – 1.53 (m, 2H), 1.52 – 1.48 (m, 1H), 1.22 (m, 2H), 1.02 (s, 3H), 0.90 (t, *J* = 11.6 Hz, 3H). ^13^C NMR (151 MHz, CDCl_3_, Supplementary Fig. 2) δ 204.67, 186.70, 174.59, 166.30, 152.30, 129.72, 125.02, 100.77, 99.60, 91.14, 81.20, 72.16, 71.91, 68.55, 48.38, 48.19, 43.99, 41.01, 36.54, 35.99, 34.15, 32.18, 31.00, 28.38, 27.35, 26.25, 26.00, 22.89, 16.50, 14.64. LRMS [C_29_H_38_FNO_9_] (+ ve ion mode) (m/z): 564 [M+1]+, 501, 481, 355.

The absolute configuration of the substituent cyclohexane in ND8008 was determined as follows. ND8008 was treated with Zn/HOAc to remove the nitrate ester, and then was hydrolysed by 2N LiOH to give the corresponding 4-hydroxycyclohexanecarboxylic acid. Through these reactions, the steric configuration of the substituent cyclohexane was unaffected. The melting point of ND8008-derived 4-hydroxycyclohexanecarboxylic acid was determined as 146.9-148.1°C (m.p. 148°C (trans), 152°C (cis)), indicating that ND8008 was the trans-isomer. The configuration of the ND8008-derived 4-hydroxycyclohexanecarboxylic acid was further confirmed by ^1^H NMR and ^13^C NMR spectrums, which were completely coincident with that of trans-4-hydroxycyclohexanecarboxylic acid (Ark Pharm, Inc., Libertyville, USA) (Supplementary Fig. 5-6). In addition, 2D NOESY spectrum was used to determine the interaction of C1’-H and C4’-H of the cyclohexyl in ND8008. As shown in Supplementary Fig. 3-4, no interaction between the two hydrogen was found.

**2 Supplementary Figures**


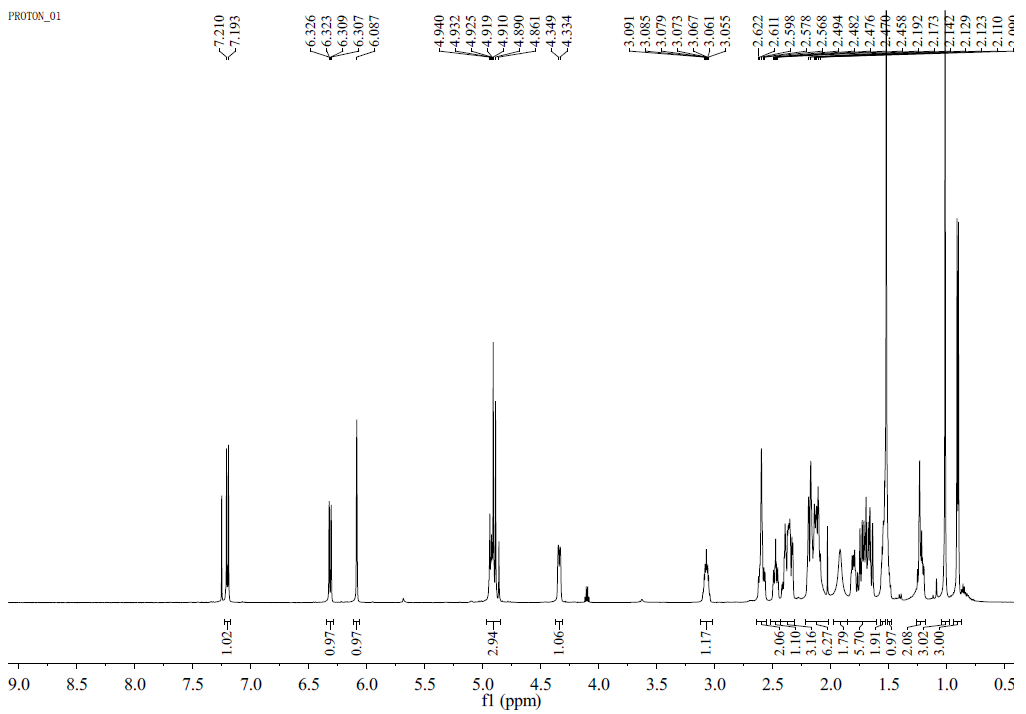


Supplementary Fig. 1 1H NMR spectra (CDCl3) of ND8008


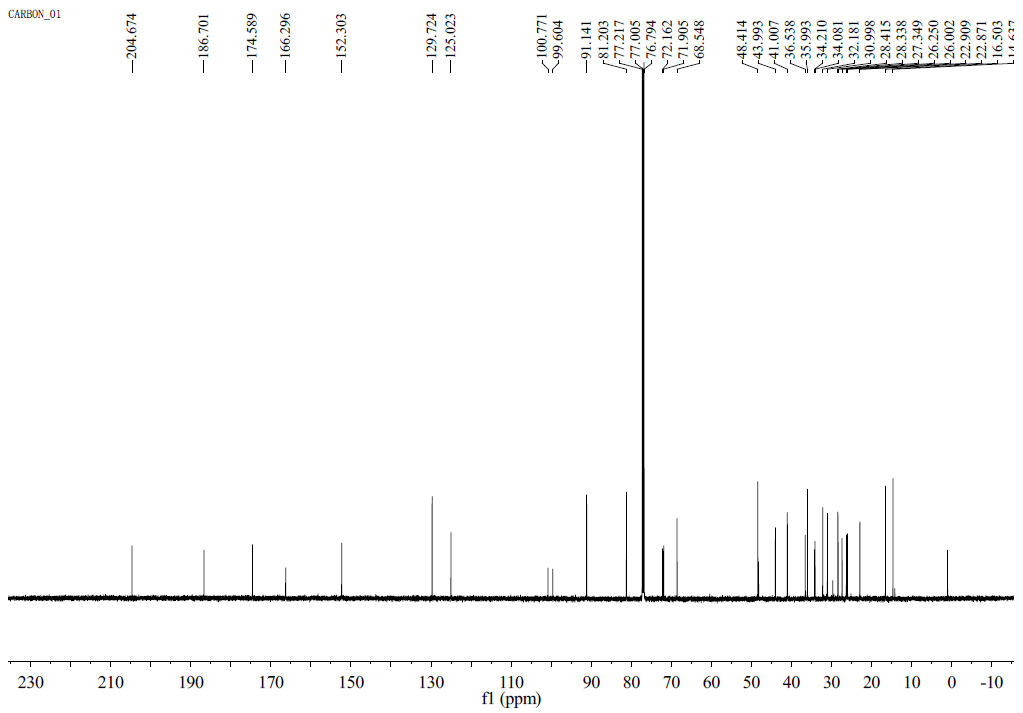


Supplementary Fig. 2: ^13^C NMR spectra (CDCl_3_) of ND8008
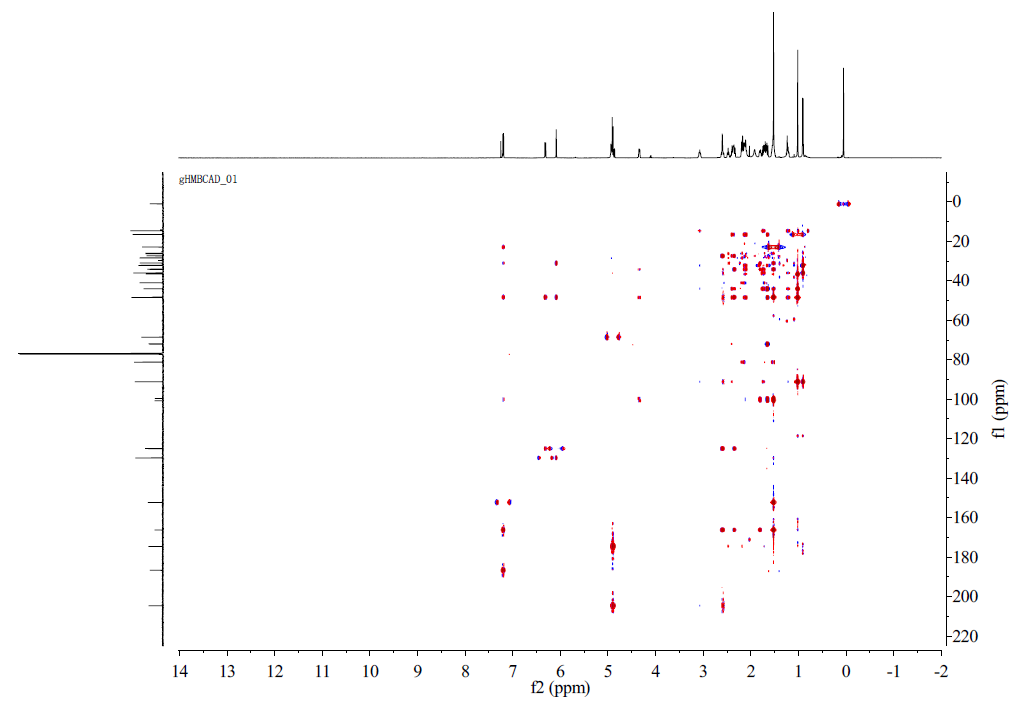


Supplementary Fig. 3: HMBC spectra (CDCl_3_) of ND8008


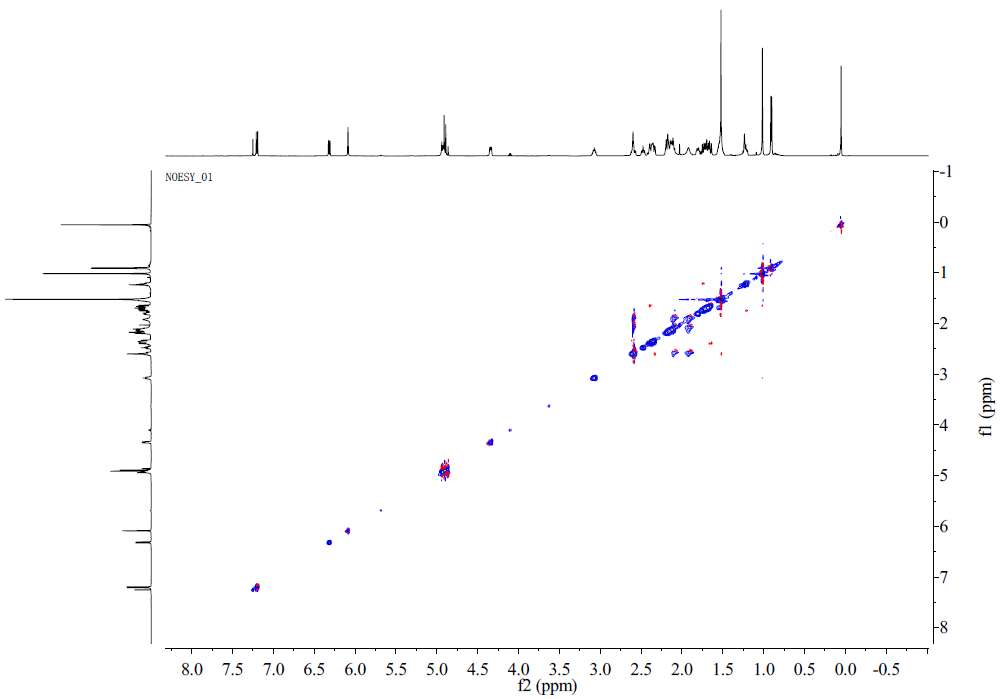


Supplementary Fig. 4: NOESY spectra (CDCl_3_) of ND8008

**
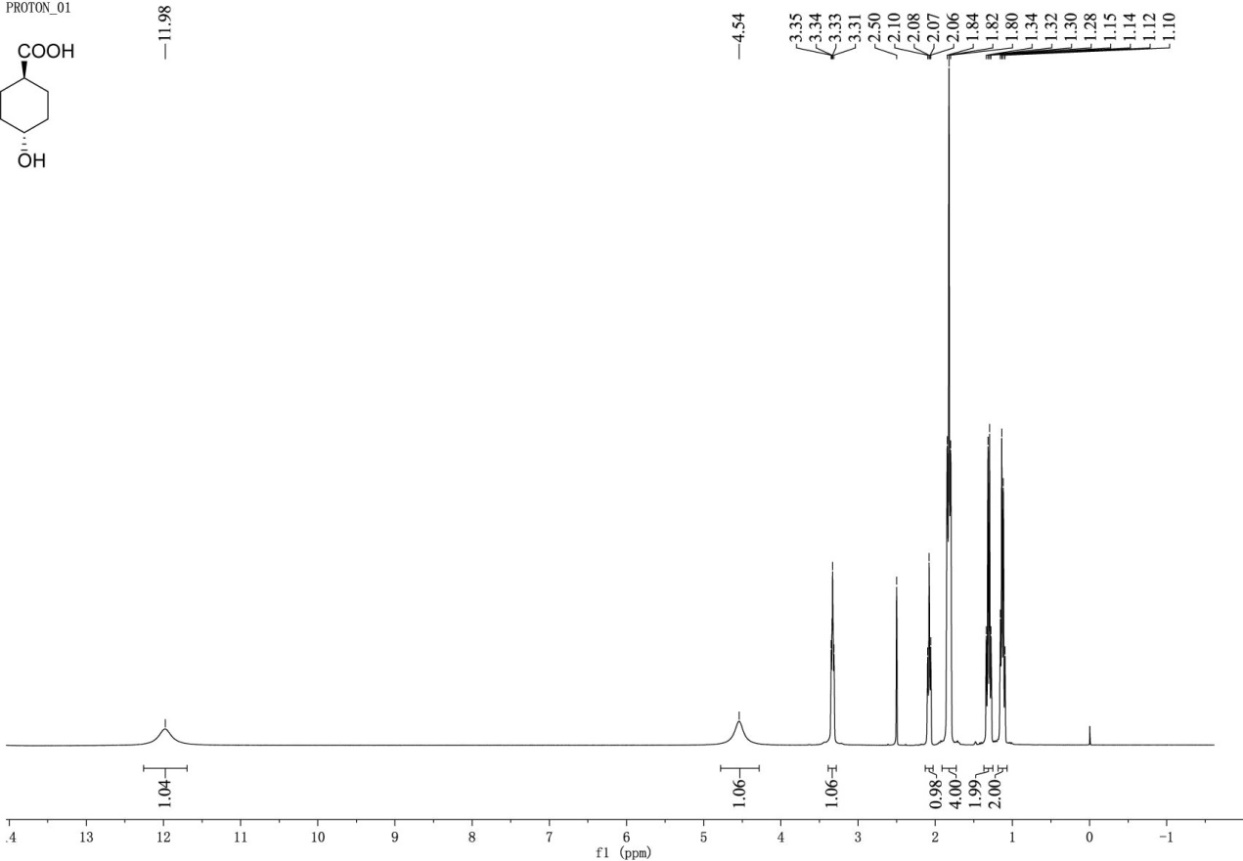
**

Supplementary Fig. 5 ^1^H NMR spectra (DMSO-d^6^) of ND8008-derived 4-hydroxycyclohexane- carboxylic acid

**
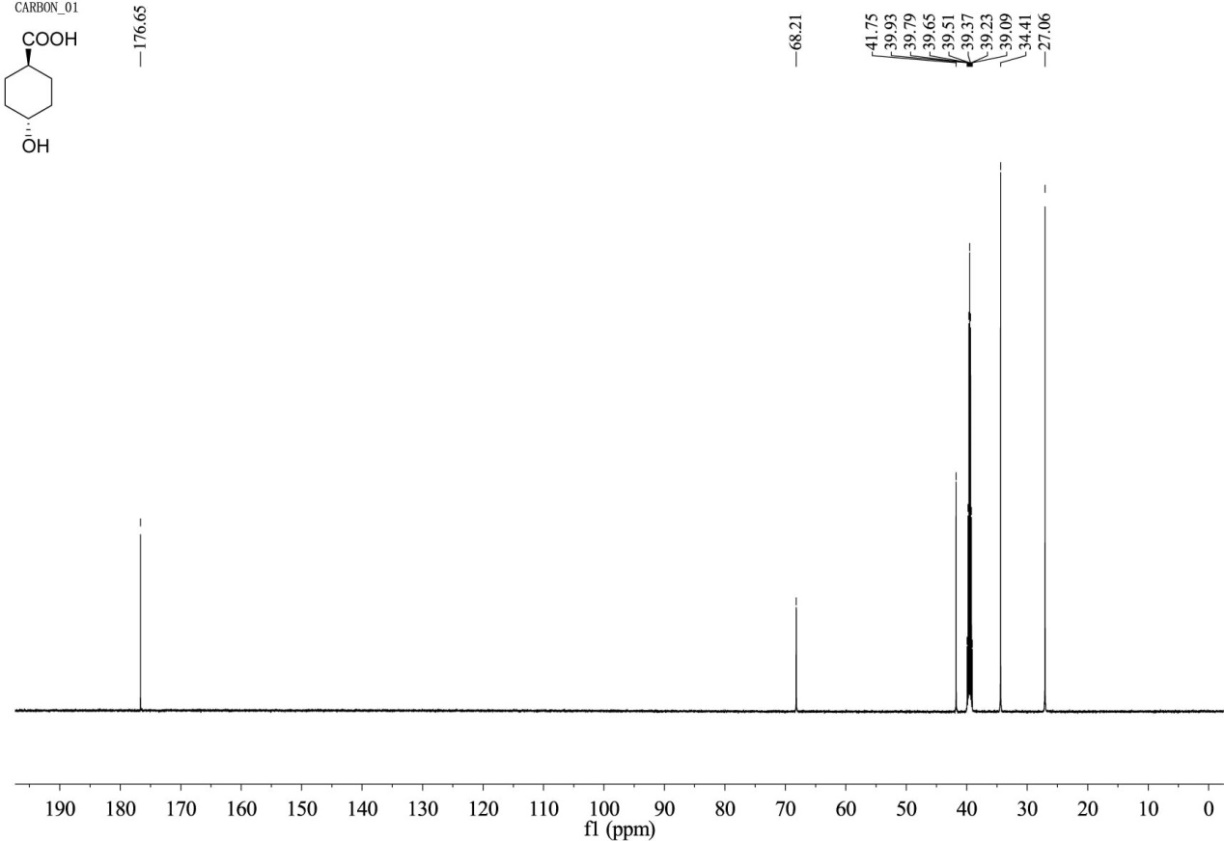
**

Supplementary Fig. 6 ^13^C NMR spectra (DMSO-d^6^) of ND8008-derived 4-hydroxycyclohexane- carboxylic acid

3 Supplementary Tables

| **Gene** | **Sequencing（5'-3'）** | **Reference** |
| --- | --- | --- |
| *gyrB*-F | CGCAGGCGATTTTACCATTA |  |
| *gyrB*-R | GCTTTCGCTAGATCAAAGTCG |
| *RNAIII*-F | GCCATCCCAACTTAATAACCA |
| *RNAIII*-R | TGTTGTTTACGATAGCTTACATGC |
| *agrA*-F | CGAAGACGATCCAAAACAAAG |
| *agrA*-R | ATGTTACCAACTGGGTCATGC |
| *icaA*-F | GGCTGCGGTAACTGGCAATCC |  |
| *icaA*-R | CTTGCCAGTTAAAGATTGGGC |

Supplementary Table S1: Sequences of the primers for Quantitative RT-PCR.

**Reference:**

1. N. R. CAMPBEL JHH. 4-Hydroxycyclohexane-1-carboxylic Acid. Journal of the Chemical Society. 1950:1379-82 doi:10.1039/JR9500001379

2. Abdelhady W, Chen L, Bayer AS, Seidl K, Yeaman MR, Kreiswirth BN et al. Early agr activation correlates with vancomycin treatment failure in multi-clonotype MRSA endovascular infections. The Journal of antimicrobial chemotherapy. 2015;70(5):1443-52. doi:10.1093/jac/dku547.

3. Huang Q, Fei J, Yu HJ, Gou YB, Huang XK. Effects of human beta-defensin-3 on biofilm formationregulating genes dltB and icaA in Staphylococcus aureus. Molecular medicine reports. 2014;10(2):825-31. doi:10.3892/mmr.2014.2309.
